# Ulcer of the Œsophagus, Possibly Cancerous in Nature

**Published:** 1882-04

**Authors:** A. D. Sturgis

**Affiliations:** Student in Veterinary Medicine


### V.—ULCER OF THE ŒSOPHAGUS, POSSIBLY CANCEROUS IN NATURE.

REPORTED BY A. D. STURGIS,
Student in Veterinary Medicine.

*Previous History.*—The cow was somewhat advanced in years, and had suffered from a cough for the past ten years or more. For the last two months she had given every evidence of being a very sick animal. At first it was noticed that whenever she attempted to eat grass or anything solid, it caused nausea, and the major part of what was swallowed was vomited. Later on water produced the same effect as the solid foods. Some of the solids and fluids, however, must have passed into the stomach and been digested.

When I first saw the animal she was very weak and emaciated, lying on the ground, where she had lain for the past forty-eight hours, unable to rise. Respirations, eight per minute; pulse imperceptible.

Owing to the extreme prostration of the patient, treatment was not considered, and it was concluded to destroy the animal.

When the throat was cut not more than two quarts of blood escaped, and that was very pale compared with that ordinarily met with.

*Necropsy Immediately after Death*—When cut open all evidence of adipose tissue had disappeared, little remaining save the skeleton.

The mouth, tongue, fauces and pharynx were carefully examined, and nothing abnormal found.

The superior portion of the œsophagus also appeared normal, but when near its junction with the stomach, a very large eroded and ulcerated spot was met with.

The ulcer in the œsophagus was fifteen centimetres in diameter. At this point the wall of the gullet was thickened, but there was no evidence whatever of a stricture. The ulcerated portion was examined microscopically at the School of Histology and Pathology, in connection with the Columbia Veterinary College and School of Comparative Medicine, by Professor Porter and William G. Le Boutillier, who rendered the following report: "Sections were made which included the apparently healthy portion of the œsophagus and the ulcerated portion. At the junction of the two the pavement epithelium of the normal mucous membrane was unusually well marked and somewhat enlarged. The ulcerated portion upon the free surface was similar to ordinary granulating tissue; its base, however, was of a dense fibrillated connective tissue substance. The muscular coat was somewhat hypertrophied, and each muscular bundle and often each muscular cell was surrounded by this dense tissue. At many points the muscle elements had dropped out and left a series of fibrous rings.

"No positive evidence of a carcinomatous structure could be detected, but some suspicious points were met with."

WILTON, CONN., March, 1882.

[The inability to swallow in this case could hardly be accredited to an organic stricture, but the irritation of food passing the eroded spot may have caused a spasmodic stricture above the ulcerated portion, and thus prevented the passage of any solid food and even fluids into the stomach. —ED.]

